# A Meta-Analysis of Cognitive Impairment and Decline Associated with Adjuvant Chemotherapy in Women with Breast Cancer

**DOI:** 10.3389/fonc.2015.00059

**Published:** 2015-03-10

**Authors:** Miyuki Ono, James M. Ogilvie, Jennifer S. Wilson, Heather J. Green, Suzanne K. Chambers, Tamara Ownsworth, David H. K. Shum

**Affiliations:** ^1^Griffith Health Institute Behavioural Basis of Health Program, School of Applied Psychology, Griffith University, Brisbane, QLD, Australia; ^2^Griffith Health Institute Behavioural Basis of Health Program, School of Applied Psychology, Griffith University, Gold Coast, QLD, Australia

**Keywords:** breast cancer, adjuvant chemotherapy, meta-analysis, cognitive functioning, moderators

## Abstract

A meta-analysis was performed to quantify the magnitude and nature of the association between adjuvant chemotherapy and performance on a range of cognitive domains among breast cancer patients. A total of 27 studies (14 cross-sectional, 8 both cross-sectional and prospective, and 5 prospective) were included in the analyses, involving 1562 breast cancer patients who had undergone adjuvant chemotherapy and 2799 controls that included breast cancer patients who did not receive adjuvant chemotherapy. A total of 737 effect sizes (Cohen’s *d*) were calculated for cross-sectional and prospective longitudinal studies separately and classified into eight cognitive domains. The mean effect sizes varied across cross-sectional and prospective longitudinal studies (ranging from −1.12 to 0.62 and −0.29 to 1.12, respectively). Each cognitive domain produced small effect sizes for cross-sectional and prospective longitudinal studies (ranging from −0.25 to 0.41). Results from cross-sectional studies indicated a significant association between adjuvant chemotherapy and cognitive impairment that held across studies with varied methodological approaches. For prospective studies, results generally indicated that cognitive functioning improved over time after receiving adjuvant chemotherapy. Greater cognitive impairment was reported in cross-sectional studies comparing chemotherapy groups with healthy control groups. Results suggested that cognitive impairment is present among breast cancer patients irrespective of a history of chemotherapy. Prospective longitudinal research is warranted to examine the degree and persisting nature of cognitive impairment present both before and after chemotherapy, with comparisons made to participants’ cognitive function prior to diagnosis. Accurate understanding of the effects of chemotherapy is essential to enable informed decisions regarding treatment and to improve quality of life among breast cancer patients.

## Introduction

Breast cancer has been reported as the second most commonly diagnosed cancer (1). Adjuvant chemotherapy increases the survival rate in breast cancer patients and is currently administered to up to 60% of patients below the age of 60 years (2). Indeed, it was reported that the 5-year survival rates after breast cancer diagnosis were 89.2% during 2004–2010, and it was estimated that almost 2.9 million women were currently living with breast cancer in the United States in 2010 (1). Hence, quality of life has become an important issue for breast cancer survivors. Although its medical efficacy is undeniable, the negative effects of adjuvant chemotherapy on cognitive functioning have been reported by some breast cancer patients, even years after treatment in some cases (3–9). To support informed decision making, it is important to understand the magnitude and specific areas of cognitive impairment that breast cancer patients may experience after adjuvant chemotherapy.

An increasing number of studies have examined the effects of adjuvant chemotherapy for breast cancer on cognitive functioning (10–13). More specifically, levels of cognitive functioning between women with a history of chemotherapy and their comparison in cross-sectional studies (i.e., termed “cognitive impairment”) and changes in levels of cognitive functioning pre- and post-chemotherapy in prospective longitudinal studies (i.e., termed “cognitive decline”) have been investigated. A recent meta-analysis suggests that breast cancer patients exposed to adjuvant therapy perform worse than comparison groups (e.g., cancer patients who do not receive adjuvant therapy, non-cancer comparison group) or normative data (11). However, these studies have not found consistent evidence of impairment within a specific neurocognitive domain. For example, neuropsychological outcomes have varied according to characteristics of the breast cancer sample studied, such as stage of tumor, time since treatment or diagnosis, menopausal status, and the use of tamoxifen or other anti-estrogen drugs, age, education level, and the amount of chemotherapy that patients received (10, 11, 14, 15). In addition, different control groups (e.g., pre-chemotherapy baseline, healthy control, or cancer control) have been used in these studies. Such inconsistencies make comparison between studies difficult since post-chemotherapy cognitive impairment may be observed only among a particular subgroup of breast cancer patients.

Furthermore, the definition of cognitive impairment/decline lacks consistency across studies. For example, it has been defined as a 1-SD decline (16), a 1.96 SDs decline (17), a 2 SDs (18) decline, or a 1.64 z-score decline (19) from pre- to post-chemotherapy. In cross-sectional studies, cognitive impairment has been typically defined as a score at least 2 SDs below the mean of a healthy control group on a test index (6, 20–23) or of the relevant published norm (24). Other studies categorized levels of impairment into mild (1 SD below on one test index) and moderate (2 SDs below on one test index) as compared to the relevant published norm (25). Cognitive impairment was also defined using the mean *z*-score of the relevant published test norm with various SDs, ranging from 1.4 SDs (26) to 2.0 SDs (27). The score at or below the fifth percentile of the control group was also used to define an overall impairment in some studies (5, 22). Consequently, evidence of post-chemotherapy cognitive impairment/decline among breast cancer patients may vary according to the definition employed in studies. Overall, it must be noted that there is no widely accepted statistical convention or cut-off in determining clinically significant declines or impairments in cognitive functioning. However, Zakzanis (28) proposed that a Cohen’s *d* effect size greater than ±3.0 is an appropriate marker of clinical significance in determining the sensitivity of neuropsychological tests.

Given the inconsistencies in the literature, the use of a single, universal unit (e.g., effect size) is ideal to synthesize findings and form a consensus on the negative effects of adjuvant chemotherapy on cognitive functioning among breast cancer patients. Indeed, four meta-analytic reviews have been conducted to date (10, 11, 14, 15). Table 1 summarizes the cognitive domains examined by each review.

**Table 1:** **Meta-analytic studies and examined cognitive domains (*k* = number of comparisons within a meta-analysis, *N* = combined number of participants)**.

Cognitive domain	Authors (reference, *K* = Study *N*)				
	Falleti et al. [(10), *K* = 6]		Jansen et al. [(13), *K* = 16]^a^	Stewart et al. [(15), *K* = 7]	Jim et al. [(11), *K* = 17]
		
	Cross-sectional	Prospective	Both cross-sectional and prospective		
Attention	*k* = 36, *N* = 330	*k* = 3	*N* = 830	*k* = 14, *N* = 366	*k* = 21
Executive function	*k* = 31, *N* = 330	*k* = 5	*N* = 996	Working memory: *k* = 15, *N* = 266	*k* = 19
Information processing speed	N/A		*N* = 617	*k* = 23, *N* = 336	*k* = 11
Motor speed/function	*k* = 12, *N* = 275	*k* = 2	*N* = 816	*k* = 16, *N* = 325	*k* = 11
Verbal ability/language	*k* = 3, *N* = 70		*N* = 795	*k* = 12, *N* = 372	*k* = 15
Visuospatial ability/skill	*k* = 5, *N* = 153	*k* = 1	*N* = 782	*k* = 10, *N* = 344	*k* = 9
Memory	*k* = 35, *N* = 330	*k* = 4	N/A	N/A	N/A
Verbal memory	N/A		*N* = 902	N/A	*k* = 23
Visual memory	N/A		*N* = 591	N/A	*k* = 21
Short-term memory	N/A		N/A	*k* = 18, *N* = 328	N/A
Long-term Memory	N/A		N/A	*k* = 21, *N* = 364	N/A

*^a^*K* = 9 focusing on breast cancer*.

The first meta-analysis published by Falleti et al. examined the nature and severity of cognitive impairment associated with adjuvant chemotherapy using five cross-sectional studies and one prospective longitudinal study (10). Analysis of cross-sectional studies revealed that the chemotherapy group performed worse than controls in all six cognitive domains (see Table 1). Of these, significant cognitive impairment was observed in the domains of spatial ability (*d* = −0.48) and language (*d* = −0.41). The authors also reported statistically significant logarithmic relationships between larger effect sizes (i.e., more significant cognitive impairment) and shorter time since last chemotherapy, greater proportions of patients currently treated with tamoxifen, and younger patient age. Younger patients may have been treated with tamoxifen more often than older patients, although this was not examined. Regardless, the results suggest that specific subsets of breast cancer patients may be more vulnerable to the cognitive effects of chemotherapy. In contrast, analysis of a prospective longitudinal study showed a wide range of positive effect sizes (i.e., improvement) across cognitive domains (*d* = 0.11 in motor function to *d* = 1.09 in attention). It was concluded that the magnitude of impairment in each domain is moderated by particular variables (e.g., age, time since last chemotherapy and chemotherapy type) and influenced by study design (cross-sectional vs. prospective). However, only one prospective longitudinal study was included in this early meta-analysis.

The aim of the second meta-analysis, published in 2005 by Jansen et al. (13), was to examine the effects of post-chemotherapy cognitive impairment among cancer patients in eight cognitive domains (see Table 1). Sixteen studies were included in this analysis and, although not all, the majority of those studies (*k* = 9) focused on breast cancer patients. It also aimed to differentiate the effect sizes by type of control data: normative data, control group data, or chemotherapy patients’ baseline data. Only visual memory showed significant impairment among chemotherapy patients across all comparison types. When the neuropsychological test scores of chemotherapy patients were compared with normative data, significant effect sizes (*d* = −0.52 to *d* = −0.78) were found in four cognitive domains (i.e., executive function, information processing speed, verbal memory, and visual memory). Conversely, a significant, but low level of impairment in language and verbal memory was identified when scores of chemotherapy patients were compared with those of healthy matched controls. However, no significant differences were identified on these domains when chemotherapy patients were compared with control patients treated with local therapy or with their own baseline scores. The analyses conducted only with breast cancer patients showed similar results (i.e., effect size, significance). Hence, the degree of impairment in each cognitive domain associated with chemotherapy varied, depending on control group characteristics. Nevertheless, the potential moderating role of control group type was not formally examined.

Stewart et al.’s (15) meta-analysis in 2006 examined seven studies (with one longitudinal), including the six examined by Falleti et al. (10). Of the eight cognitive domains evaluated (see Table 1), statistically significant small to medium weighted pooled effect sizes (*d* = −0.24 to −0.37) were found in all domains except simple attention and processing speed. The largest effect sizes were found in language (*d* = −0.37) and short-term memory (*d* = −0.31). However, the fail-safe numbers were smaller than recommended. It was concluded that cognitive impairment was subtle and/or only seen among a particular subgroup of women. The authors did not differentiate the effect sizes by type of control group or study design, and this may explain the relatively smaller grand mean effect sizes found in this review than those found in previous reviews. In addition, in this meta-analysis studies were manually removed from analyses for each cognitive domain to achieve homogeneity. Thus, the results may not be representative of the broader breast cancer population.

In the most recent meta-analytic review by Jim et al. in 2012 (11), cognitive functioning in the post-treatment period (i.e., at least 6 months post-therapy) among breast cancer patients was examined. It also examined demographic and clinical moderators of cognitive impairment in patients with breast cancer, including age, education, time since chemotherapy, and treatment with endocrine therapy. The authors included 17 studies, which varied in type of control group: patients’ pre-chemotherapy baseline (*k* = 4); patients who received local therapy (i.e., radiation, surgery) or endocrine therapy (*k* = 6); patients without cancer (*k* = 3); two types of control group (pre-chemotherapy baseline and local or endocrine therapy only, *k* = 2); and all three types of control group (*k* = 2). Overall, chemotherapy patients performed worse in the domains of verbal ability (*g* = −0.19, *p* < 0.01) and visuospatial ability (*g* = −0.27, *p* < 0.01). As post-chemotherapy cognitive impairment in these domains depended on types of comparisons (i.e., type of control group), type of control group was reported as a likely moderating factor, although this was not formally tested. Thus, it remains unclear whether the type of control group significantly moderates the magnitude of post-chemotherapy cognitive impairment among breast cancer patients. In addition, no demographic or clinical factors were found to moderate observed cognitive impairment in verbal ability or visuospatial ability (all *p* > 0.05). This may be partly due to their inclusion criteria being at least 6 months post-treatment where any cognitive impairment experienced may have diminished with time. Alternatively, as significant moderators were reported by Falleti et al. (10), results may need to be analyzed separately by study design (cross-sectional vs. prospective longitudinal studies). In the current meta-analysis, moderating factors are examined for cross-sectional studies and prospective longitudinal studies separately.

While there is a general consensus in these meta-analytic reviews regarding the adverse effects of chemotherapy on cognitive functioning among breast cancer patients, their specific findings varied. For example, while some cognitive domains (e.g., language) have more consistently been identified as affected functions, the results have not been firmly conclusive. This may be due to the small number of studies included, and/or a strict inclusion criteria employed, that is, at least 6 months post-treatment in Jim et al. (11). In addition, it has been suggested that grand mean effect sizes may obscure the detection of subtle cognitive decline in a vulnerable subgroup (10, 27). Hence, identification of factors that moderate the magnitude of post-chemotherapy cognitive impairment is important. Indeed, as discussed above, Falleti et al. (10) reported moderators (e.g., time since treatment, younger age, current tamoxifen use), but these results were inconsistent with Jim et al.’s (11) results. Furthermore, although suggested (11, 14), the moderating role of type of controls has never been tested formally.

Some studies (3, 17, 29, 30) have reported that psychological factors such as fatigue, depression, and anxiety can have a negative impact on cognitive functioning in cancer patients. Previous studies that examined the role of chemotherapy in cognitive functioning typically either excluded breast cancer patients with past and/or current psychiatric disorders (5, 8, 17, 18, 20–24, 27, 31–36), found no significant group differences in emotional functioning (9, 12, 37), or statistically controlled for these factors (7, 26, 38). Indeed, the role of psychological factors in post-chemotherapy cognitive functioning was not examined in pervious meta-analyses. Consequently, the current meta-analysis did not include psychological factors as moderating factors.

The current meta-analytic review includes a broader selection of studies compared to previous reviews with two study aims. First, it aimed to identify the magnitude of cognitive impairment among breast cancer patients treated with adjuvant chemotherapy in eight cognitive domains: attention; executive function; long-term (delayed) memory; short-term memory; speed of processing; language; visuospatial; and motor function. The selection of domains was based on clinical practice and neuropsychological assessment literature (39–41). The categories of short- and long-term memories were deemed more appropriate than verbal and visual memories, given that the effect of chemotherapy is more global or diffuse in nature rather than localized in one hemisphere (42). Second, this review aimed to identify factors that moderate the magnitude of cognitive impairment among breast cancer patients treated with chemotherapy. As discussed previously, the findings of moderating factors in previous meta-analyses have been mixed. However, this may be partly because cross-sectional studies and prospective longitudinal studies have different study focuses, i.e., cognitive impairment and cognitive decline, respectively. Indeed, study design (e.g., cross-sectional vs. prospective) has been suggested to moderate the results (10, 11). Thus, the moderating effects of time since treatment, type of control group, and patients’ demographic characteristics (age and education level) were examined separately for cross-sectional studies and prospective longitudinal studies via meta-regression. Identification of moderators would advance knowledge of risk factors for experiencing cognitive impairment associated with chemotherapy among breast cancer patients.

## Method

### Search strategy

Three search strategies were employed to identify suitable published studies for inclusion in the meta-analysis. First, nine computerized databases were searched: PsychINFO, ProQuest Psychology, PsycARTICLES, PubMed/MEDLINE, CINAHL, Web of Science ISI, Scopus, Cambridge Scientific Abstracts, and Google Scholar. The keywords used to search the databases included: *breast cancer, breast neoplasms, chemotherapy, adjuvant chemotherapy, treatment effects, cognition, cognitive, cognitive functioning, neurocognitive, neuropsychological, neuropsychological tests, cancer treatment*, and *cognitive impairment*. Second, the reference lists of published studies collected, and previous meta-analyses and narrative reviews of the topic (10, 11, 14, 15) were scanned to locate further studies not found in the database searches. Third, manual searches of relevant journals were conducted to identify studies, including Clinical Breast Cancer, Journal of Clinical Oncology, Cancer, Journal of Neuro-Oncology, Neuropsychologia, Journal of Clinical and Experimental Neuropsychology, Psycho-Oncology, Neuro-Oncology, Neuro-Oncology Practice, and Acta Oncologia. The search was inclusive of studies published up to August 2014.

### Inclusion criteria

To be included in the meta-analysis, studies had to satisfy the following criteria:

1. Studies report objective neuropsychological data regarding women with breast cancer who underwent adjuvant chemotherapy using either cross-sectional (i.e., comparison groups) or prospective (i.e., patients assessed before and after chemotherapy) designs;
2. For cross-sectional studies, the comparison group consisted of healthy individuals or breast cancer patients not receiving chemotherapy (e.g., local therapy only);
3. For prospective longitudinal studies, patients were assessed before the commencement of chemotherapy and at least one time point after the completion of chemotherapy;
4. At least one validated measure of neuropsychological functioning was used. Studies reporting data from screening measures only (e.g., Mini Mental Status Exam, High Sensitivity Cognitive Screen) were excluded;
5. The results were published in a peer-reviewed journal and in English;
6. Each study reported original group data – the data did not relate to individual case-studies, reviews, commentaries or meta-analyses; and
7. The results presented were sufficient to calculate effect sizes (i.e., means and SDs, *t*-values, *F*-values, *p*-values, or *r*-values).

Data extracted from studies included neuropsychological test data (i.e., mean scores, SDs, and sample size), study design characteristics (i.e., type of control group and timing of assessments), and participant characteristics (i.e., age, education, intelligence assessment, and time since chemotherapy). When sufficient information was not present to calculate effect sizes, an attempt was made to contact authors to obtain the required information. Nineteen authors were contacted to obtain additional information, and one author replied with sufficient data.

### Study design and classification

**Figure 1:**
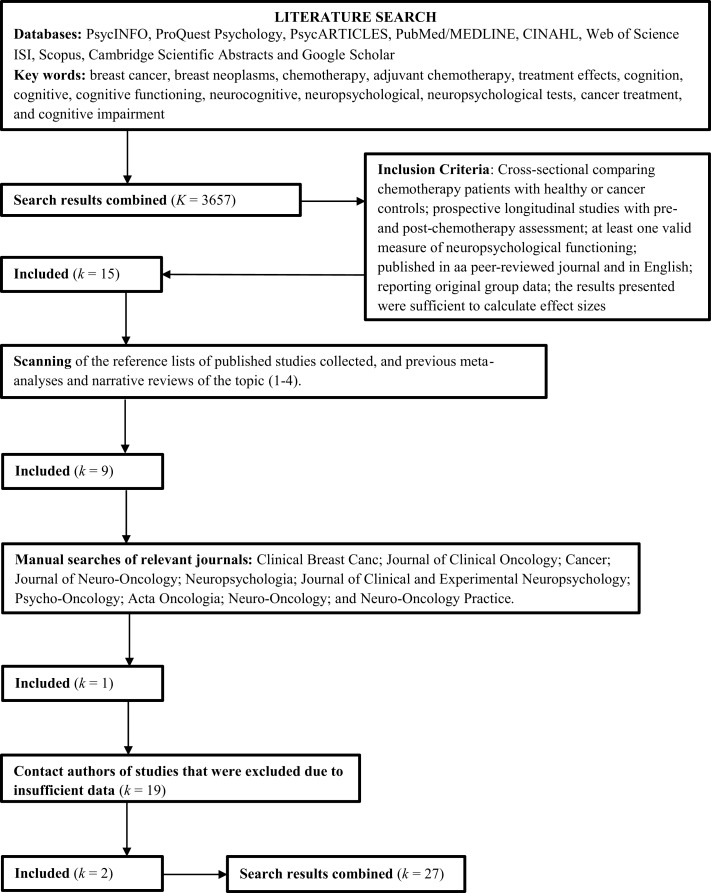
**Flow diagram: literature search and selection**.

As shown in Figure 1, a total of 27 studies were included in the meta-analysis, including 14 reporting cross-sectional data only, eight reporting both cross-sectional and prospective data, and five reporting prospective data only.

Effect sizes were calculated separately for cross-sectional and prospective designs. For studies reporting cross-sectional data, samples were grouped according to treatment type and dosage and comparison groups. Two studies (5, 26) included two groups of chemotherapy patients of standard-dose and high-dose chemotherapy, and one study (38) included two groups of chemotherapy patients, namely, those receiving chemotherapy alone and those receiving chemotherapy and tamoxifen. For these studies, two sets of effect sizes were calculated, one for each chemotherapy group contrasted against the comparison group. Four studies (33, 34, 38, 43) included cross-sectional data on cognitive functioning at multiple time points after the completion of chemotherapy [e.g., 5–6 months and 1 year follow-up for Collins et al. (33)] for chemotherapy and comparison groups. For these studies, a set of effect sizes was calculated at each time point, with time after completion of chemotherapy recorded for analyses of moderators. For cross-sectional studies included in the meta-analysis, a range of comparison groups were used to compare cognitive functioning of chemotherapy patients. Comparison groups included healthy controls (12 studies), breast cancer patients not receiving any treatment (one study), breast cancer patients receiving adjuvant endocrine/hormonal treatment only (four studies), and breast cancer patients receiving local therapy (i.e., surgery and/or radiation) only (11 studies). The type of comparison group (i.e., healthy vs. patient comparisons) was examined as a potential moderator of effect sizes using a meta-analytic regression random effects model (44, 45). Four cross-sectional studies (8, 32, 37, 43) included two comparison groups (i.e., healthy controls and patient controls) to contrast the cognitive functioning of patients receiving chemotherapy. For these studies, two sets of effect sizes were calculated, one for each control group contrasted against the chemotherapy group.

### Neuropsychological measures

A total of 81 independent neuropsychological measures were used across the studies included in the meta-analysis. These neuropsychological measures were categorized into eight separate cognitive domains according to the primary cognitive function each test is purported to assess based on clinical practice and neuropsychological assessment literature (39–41). Table 2 displays the eight cognitive domains and the individual neuropsychological measures assigned to each category. Although a single neuropsychological measure may tap multiple cognitive functions, an effort was made to assign each individual measure to a single cognitive domain according to a primary domain of cognitive functioning as specified by major test compendiums. This approach was adopted to minimize over-inflation and violation of the independence of mean effect sizes in the meta-analysis. Tests of homogeneity of effect sizes were performed within each domain of cognitive functioning to assess whether the neuropsychological tests were measuring common parameters.

**Table 2:** **Cognitive domains assigned to the neuropsychological measures**.

Cognitive domain	Neuropsychological measures^a^
Attention	Arithmetic (WAIS),^2,5,17,22,25,27^ CNS-vital signs (flexibility, working memory),^6^ continuous performance test (CPT),^1,2^ D2 test (GZ-F),^11,18,19,23^ digit span (forwards and backwards, WAIS and WMS),^2,5,6,10,11,14,15,17,18,19, 21,22,23,24,25,26,27^ digit symbol (WAIS),^1,4,5,6,7,9,10,11,12,15,18,22,23,25,26^ paced auditory serial addition test (PASAT),^4,5,6,22^ RBANS attention,^13^ spatial span (WAIS and WMS),^5,10, 14,15, 22^ test of everyday attention (TEA; auditory elevator^9^, telephone search^24^), test battery for attentional performance (TAP; Alertness,^19^ Go/No-Go^19^), trail making test A,^1,4,5,6,7,9,10,11,12,15,17,18, 19,20,22,23,25,26,27^ visual elevator,^24^ visual span (WAIS),^19^ and visual attention test^20^
Executive function	Consonant trigrams,^5,22^ controlled oral word association,^6,17,26^ D-KEFS Sorting Task,^24^ IED Stage 5,^17,27^ Regensburg word fluency test (RWT),^11,19^ trail making test B,^1,4,5, 6,7,10,11,12,15,17,18,19,20,22,23,25,26,27^ stroop color-word,^4,7, 8,12,13,14,16,18,20,21,23,24^ verbal fluency,^1,4,5, 6,7,10,11,12,15,16,18,19,20,22,23,24,25^ and Wisconsin Card Sorting Test^5,17,22,27^
Long-term memory	15-Word learning test (delayed and recognition),^16^ Benton facial recognition test,^17,27^ Benton visual retention total errors,^17,27^ brief visuospatial memory test (BVMT) revised (delayed),^6^ California verbal learning test (delayed recall and recognition),^1,4,5,7,10,15,22^ CNS-vital signs (visual and verbal, delayed),^6^ family pictures (WMS; delayed and recognition),^2,5,22^ logical memory (WMS; delayed and recognition),^1,4,5,11,14,21,22^ Hopkins verbal learning test revised (delayed recall),^6,12,26^ RBANS delayed memory,^13^ Rey auditory verbal learning test (delayed recall and recognition),^3,5,14,17, 18,19,21, 22,23, 24,27^ Rey complex figure test (delayed recall and recognition),^3,4,12,14,17,19,20,21, 23,27^ visual verbal learning test (delayed and total),^8^ and visual reproduction (WMS; delayed and recognition)^1,7,10,15,18, 20,24^
Short-term memory	4WSTM,^3^ 15-word learning test (immediate recall),^16^ auditory consonant trigrams test,^6^ Benton visual retention test revised,^6,17,27^ BVMT revised (total),^6^ California verbal learning test (immediate recall),^1,4,5,7,15,22^ CNS-vital signs (visual and verbal, immediate),^6^ Hopkins verbal learning test revised (total),^6,26^ letter digit coding test,^8^ letter digit substitution test,^16^ letter-number sequencing (WAIS),^2,5,6,14,17,20,21, 22,27^ logical memory (WMS; immediate),^1,4,11,14^ RBANS immediate memory,^8,13^ Rey auditory verbal learning test (immediate recall),^3,5,14,17,19,20,21,22,23^ Rey complex figure test (immediate recall),^3,12,14,18, 21^ and visual reproduction (WMS; immediate)^1,4,7,10, 15,18,20,24^
Speed of processing	2 and 7 test,^15^ Bourdon-Wiersma dot Cancelation test,^9^ CNS-vital signs (processing speed, reaction time),^6^ Fepsy (binary choice, visual reaction, and visual searching),^18,23^ letter cancellation,^14,21^ letter digit substitution test,^16^ reaction time,^4,20^ symbol digit modalities test,^24^ symbol search (WAIS),^5,6,22^ and test battery for attentional performance (TAP; simple reaction time)^4,19^
Language	Boston Naming Test^1,5,12,17,22,27^, RBANS Language^8, 13^, Reading Subtest (WRAT-R)^1,17^, Vocabulary (WAIS, WASI)^1,2,12,17^, Similarities (WAIS-R, WASI)^2,17,25^
Visuospatial	Block design (WAIS, WASI),^1,2,4,5,12,17,22,25^ design organization test,^16^ matrix reasoning (WAIS, WASI),^2,17,24^ novel image/novel location,^2^ RBANS visual construction,^8,13^ and Rey complex figure test (copy)^4,12, 17,18,23, 27^
Motor function	California computerized assessment package simple reaction time,^4^ choice reaction time,^4^ Fepsy finger tapping test,^1,7,10,18,20,23^ and Perdue Grooved Peg Board^2,5,9,8,13,16,22,24,25^

*^a^Columns includes neuropsychological measures and studies that employed the measure where: 1, Ahles et al. (12); 2, Ayala-Feliciano et al. (31); 3, Bender et al. (38); 4, Castellon et al. (32); 5, Collins et al. (33); 6, Collins et al. (34); 7, de Ruiter et al. (20); 8, Debess et al. (37); 9, Deprez et al. (21); 10, Donovan et al. (24); 11, Hermelink et al. (25); 12, Hurria et al. (46); 13, Jansen et al. (35); 14, Jenkins et al. (43); 15, Jim et al. (36); 16, Koppelmans et al. (7); 17, Nguyen et al. (8); 18, Schagen et al. (22); 19, Scherwath et al. (26); 20, Schilder et al. (23); 21, Shilling et al. (19); 22, Stewart et al. (18); 23, van Dam et al. (5); 24, Vearncombe et al. (17); 25, Wefel et al. (27); 26, Wefel et al. (47); 27, Yamada et al. (9)*.

### Data collection and effect size protocol

Twenty-seven studies met inclusion criteria for the meta-analysis. The following approach was adopted to calculate effect sizes:

1. Calculation of individual effect sizes (*d*) and corresponding variances for each neuropsychological test outcome in each study. For cross-sectional studies, this was the difference between chemotherapy and control group scores, and for prospective longitudinal studies, this was the difference between pre- and post-chemotherapy scores;
2. Calculation of weighted mean effect size for each study using fixed and random effects models;
3. Calculation of weighted mean effect sizes for each cognitive domain across studies using fixed and random effects models;
4. Calculation of 95% confidence intervals (CIs) surrounding the two classes of weighted mean effect sizes (i.e., study and cognitive domain); and
5. Calculation of *Q* and *I*^2^ statistics to assess heterogeneity of weighted mean effect sizes by cognitive domain and study weighted mean effect sizes.

Cohen’s *d* (48) standardized mean difference effect sizes using pooled SDs and corrected for small sample bias (i.e., Hedge’s *g*) were used to determine the magnitude of difference in performance of neuropsychological measures. Zakzanis (28) proposed that Cohen’s *d* is the most appropriate measure for neuropsychological research primarily due to its ability to explicitly account for the variability observed between neuropsychological patients. Poorer cognitive functioning by chemotherapy groups was represented by negative effect sizes. Cohen (48) defines a small effect size as *d* ≥ 0.2, a moderate effect as *d* ≥ 0.5, and a large effect as *d* ≥ 0.8. Zakzanis (28) proposed that a Cohen’s *d* of >0.30 is an appropriate marker of clinical significance in neuropsychological functioning. All Cohen’s *d* statistics are expressed in SD units.

Both fixed and random effect models for combined summary effect sizes were computed. For fixed effect models, it is assumed that the true effect size is constant across all studies (e.g., cognitive impairment constant regardless of participant characteristics or cognitive domain), with variation being due to sampling error. For random effect models, it is assumed that the true effect size varies across studies due to known and unknown factors (e.g., participant characteristics, cognitive domain assessed).

Individual effect sizes were first calculated for every neuropsychological measure used by a study. For cross-sectional studies reporting means and SDs for neuropsychological test scores, *d* (Eq. 1) was calculated by subtracting the chemotherapy group mean score (*X*_1_) from the comparison group mean score (*X*_2_) and dividing the result by the pooled SD (*S*_pooled_) (Eq. 2). *N*_1_ is the number of participants in the chemotherapy group, *N*_2_ is the number of participants in the comparison group, SD_1_ is the SD of the mean score for the chemotherapy group, and SD_2_ is the SD of the mean score for the control group:

(1) $$d=\frac{\left(\bar{X_{2}}-\bar{X_{1}}\right)}{S_{\mathrm{pooled}}}$$

where

(2) $$S_{\mathrm{pooled}}=\sqrt{\frac{\left(N_{1}-1\right){\mathrm{SD}}_{1}^{2}+\left(N_{2}-1\right){\mathrm{SD}}_{2}^{2}}{\left(N_{1}-1\right)+\left(N_{2}-1\right)}}$$

Similarly, for prospective longitudinal studies reporting means and SDs for neuropsychological scores, *d* was calculated using Eqs 1 and 2, subtracting the post-chemotherapy mean score (*X*_2_) from the pre-chemotherapy mean score (*X*_1_) and dividing the result by the pooled SD. *N*_1_ is the number of participants pre-chemotherapy, *N*_2_ is the number of participants post-chemotherapy, SD_1_ is the SD of the mean score pre-chemotherapy, and SD_2_ is the SD of the mean score post-chemotherapy.

All computed effect sizes were corrected for small sample bias (Hedges *g*) using the formula provided by Hedges (49) and displayed in Eq. 3. *N* is the total number of participants and *d^’^* is the unbiased standardized mean difference:

(3) $$d^{\prime }=d\left[1-\frac{3}{4N-9}\right]$$

The variance for each individual effect size (*v*_d_) was calculated using Eq. 4, with *N* being the sample size for each group in cross-sectional studies and *N* being the sample size at each assessment point in prospective longitudinal studies:

(4) $$v_{d}=\left[\frac{N_{1}+N_{2}}{N_{1}N_{2}}+\frac{{\left(d^{\prime }\right)}^{2}}{2\left(N_{1}+N_{2}\right)}\right]$$

The inverse of the sampling variance (Eq. 5) was used to weight each effect size for the fixed effect model of analysis, while the inverse of the sampling variance plus a random effects variance constant (τ_θ_) was used to weight each effect size for the random effect model of analysis (Eq. 6):

(5) $$w_{i}=\frac{1}{v_{i}}$$

(6) $$w_{i}=\frac{1}{v_{i}+\tau_{\theta }}$$

where

(7) $$\tau_{\theta }=\frac{Q_{T}-\left(k-1\right)}{\sum w_{i}-\left(\frac{\sum w_{i}^{2}}{\sum w_{i}}\right)}$$

After calculation of individual effect sizes, two classes of weighted mean effect sizes $\left(\bar{d}\right)$ were calculated (steps 2 and 3 of the effect size protocol) for (1) studies and (2) cognitive domain. A mean effect size was calculated for each study by averaging all effect sizes and inverse variance weights within the study. Therefore, each study produced an average effect size and an average inverse variance weight. An average inverse variance weight was used for studies, as weights are a function of sample size and highly similar across effect sizes within a study. Weighted mean effect sizes for cognitive domain were calculated from the individual effect sizes using the formula provided by Hedges and Olkin (44). In Eq. 8, *k* is the number of effect sizes, *w*_i_ = 1/*v_i_* (inverse variance weight), and *v*_i_ is the variance of the individual effect size:

(8) $$\bar{d}=\left[\frac{\sum_{i-1}^{k}w_{i}d_{i}}{\sum_{i-1}^{k}w_{i}}\right]$$

The variance of the weighted mean effect size was then calculated using Eq. 9, which was then used to calculate 95% CIs for weighted mean effect sizes to aid in the determination of statistical significance (Eq. 10):

(9) $$v_{\bar{d}}=\left[\frac{1}{\sum_{i-1}^{k}w_{i}}\right]$$

(10) $$\text{95\% CI}=\bar{d}\pm 1.96\sqrt{v_{\bar{d}}}$$

Tests of the homogeneity of the two classes of weighted mean effect sizes were performed to determine whether the effect sizes were assessing common parameters. When the variation of effect sizes is greater than that would be expected from sampling error alone, the distribution of effect sizes is deemed to be heterogeneous and not representative of a common parameter (45). The *Q*-statistic was calculated as a homogeneity test (Eq. 11):

(11) $$Q=\sum_{i=1}^{k}w_{i}{\left(d_{i}-\bar{d}\right)}^{2}$$

where *k* is the number of effect sizes, *w_i_* is the inverse variance weight of each individual effect size, *d_i_* is the individual effect size, and $\bar{d}$ is the weighted mean effect size. If the *Q*-statistic exceeds a critical value associated with a pre-determined alpha level (in the present study, *p* < 0.05) the sample of effect sizes is characterized as heterogeneous.

A number of variables were examined that may potentially moderate the association between chemotherapy and cognitive impairment using meta-analytic regression, including time since last chemotherapy treatment, type of control group, intelligence, and patients’ average age at chemotherapy treatment. Weighted mean study effect sizes were used for all moderator analyses, which were performed separately for cross-sectional and prospective longitudinal studies. All moderators were examined as between-study variables impacting on effect size magnitude and performed separately for cross-sectional and prospective longitudinal studies. Finally, Duval and Tweedie’s (50) trim-and-fill method was used to explore publication bias.

## Results

### Participants

The 27 included studies comprised a total of 1562 breast cancer patients who received chemotherapy and 2799 comparison individuals. The mean age of the chemotherapy and comparison sample was 53.24 years (SD = 8.05) and 55.28 years (SD = 9.37), respectively. For the 16 studies reporting education as a continuous outcome, the mean years of education for the chemotherapy and comparison sample was 14.16 (SD = 1.18) and 14.37 (SD = 1.46), respectively. Previous studies typically reported that participants’ age and education level were matched between groups. For the 19 studies reporting data on intelligence, the mean IQ for the chemotherapy and comparison sample was 108.79 (SD = 4.46) and 108.13 (SD = 5.79), respectively. There was no significant difference in mean IQ scores between chemotherapy and comparison groups using a paired-samples *t*-test, *t*(14) = 0.42, *p* > 0.05.

### Mean study effect sizes

A total of 737 individual effect sizes for neuropsychological measures were calculated across all studies, with these effect sizes used to calculate a weighted mean effect size for each study and cognitive domain. Calculated effect sizes for each neuropsychological measure are available on request.

### Cross-sectional studies

Weighted mean effect sizes for cross-sectional studies using fixed and random effect models are shown in Table 3. Mean effect sizes ranged from −1.22 to 0.62 using the more conservative random effect model, with 11 comparisons from eight studies producing positive mean effect sizes (i.e., chemotherapy patients exhibited *better* overall cognitive functioning in contrast to comparison groups). Of these, six comparisons showed a significant positive effect size, and they compared cognitive functioning between breast cancer patients with and without chemotherapy (e.g., chemotherapy vs. local therapy).

**Table 3:** **Weighted mean effect sizes for cross-sectional studies**.

Study: authors, reference, comparison group	*k*	Fixed effect model				Random effect model			
		Effect size (SE)	95% CI	*z*	*Q*	Effect size (SE)	95% CI	*z*	*Q*
Grand mean weighted effect size	509	−0.12 (0.01)*	−0.14 to −0.10	− 13.40	2519.48*	−0.14 (0.02)*	−0.18 to −0.09	− 6.38	857.54*
Ahles et al. (12), local therapy only	24	−0.16 (0.05)*	−0.25 to −0.07	− 3.31	18.67	−0.16 (0.10)	−0.35 to 0.03	−1.69	4.79
Ayala-Feliciano et al. (31), healthy comparison	10	−1.12 (0.11)*	−1.34 to −0.90	− 10.01	85.92*	−1.22 (0.17)*	−1.55 to −0.89	− 7.29	39.97*
Bender et al. (38), chemotherapy only vs. patients without tamoxifen and chemotherapy, 1 week follow-up	7	0.56 (0.17)*	0.23 to 0.88	3.33	66.68*	0.62 (0.22)*	0.19 to 1.06	2.80	41.11*
Bender et al. (38), chemotherapy and tamoxifen vs. patients without tamoxifen and chemotherapy, 1 week follow-up	7	0.32 (0.17)	−0.02 to 0.65	1.87	47.28*	0.35 (0.22)	−0.09 to 0.79	1.57	29.17*
Bender et al. (38), chemotherapy only vs. patients without tamoxifen and chemotherapy, 1 year follow-up	7	−0.42 (0.24)	−0.89 to 0.06	−1.73	70.43	−0.58 (0.29)*	−1.14 to −0.01	− 2.01	56.54*
Bender et al. (38), chemotherapy and tamoxifen vs. patients without tamoxifen and chemotherapy, 1 year follow-up	7	−0.63 (0.28)*	−1.18 to −0.08	− 2.26	94.89*	−0.68 (0.32)*	−1.30 to −0.05	− 2.13	75.95
Castellon et al. (32), local therapy only	20	−0.39 (0.07)*	−0.51 to −0.26	− 5.95	19.08	−0.39 (0.11)*	−0.61 to −0.17	− 3.53	6.62
Castellon et al. (32), healthy comparison	20	−0.23 (0.06)*	−0.35 to −0.11	− 3.71	19.44	−0.23 (0.11)*	−0.45 to −0.02	− 2.15	6.43
Collins et al. (33), hormonal therapy only, one month follow-up	22	0.11 (0.04)*	0.02 to 0.20	2.51	18.29	0.11 (0.10)	−0.08 to 0.30	1.15	3.84
Collins et al. (33), hormonal therapy only, one year follow-up	22	0.01 (0.04)	−0.07 to 0.10	0.33	23.15	0.02 (0.10)	−0.17 to 0.20	0.17	4.97
Collins et al. (34), healthy comparison	12	−0.33 (0.06)*	−0.44 to −0.21	− 5.57	14.53	−0.33 (0.13)*	−0.58 to −0.08	− 2.58	3.09
de Ruiter et al. (20), without chemotherapy	15	−0.21 (0.09)*	−0.38 to −0.04	− 2.40	6.43	−0.21 (0.13)	−0.47 to 0.05	−1.56	2.70
Debess et al. (37), local therapy only	4	0.13 (0.12)	−0.10 to 0.35	1.10	4.51	0.13 (0.22)	−0.30 to 0.55	0.59	1.28
Debess et al. (37), healthy comparison	4	−0.09 (0.06)	−0.20 to 0.03	−1.42	2.92	−0.09 (0.19)	−0.46 to 0.29	−0.45	0.29
Deprez et al. (21), healthy comparison	4	−0.83 (0.17)*	−1.17 to −0.49	− 4.77	0.52	−0.83 (0.25)*	−1.32 to −0.33	− 3.29	0.25
Donovan et al. (24), local therapy only	11	0.08 (0.05)	−0.01 to 0.18	1.66	7.94	0.08 (0.13)	−0.17 to 0.33	0.64	1.17
Jenkins et al. (43), healthy comparison, four weeks follow-up	13	−0.13 (0.05)*	−0.22 to −0.03	− 2.63	6.68	−0.13 (0.12)	−0.36 to 0.11	−1.06	1.08
Jenkins et al. (43), local therapy only, four weeks follow-up	13	0.11 (0.05)*	0.01 to 0.21	2.19	6.26	0.11 (0.12)	−0.13 to 0.35	0.91	1.08
Jenkins et al. (43), healthy comparison, one year follow-up	13	−0.13 (0.05)*	−0.22 to −0.03	− 2.66	19.20	−0.13 (0.12)	−0.36 to 0.11	−1.06	3.17
Jenkins et al. (43), local therapy only, one year follow-up	13	0.15 (0.05)*	0.05 to 0.25	3.03	9.08	0.15 (0.12)	−0.08 to 0.39	1.27	1.58
Jim et al. (36), healthy comparison	13	−0.75 (0.05)*	−0.83 to −0.66	− 16.45	1071.00*	−0.74 (0.12)*	−0.98 to −0.50	− 6.15	326.39*
Koppelmans et al. (7), healthy comparison	15	−0.11 (0.02)*	−0.15 to −0.07	− 5.62	20.22	−0.11 (0.10)	−0.31 to 0.10	−1.03	0.69
Nguyen et al. (8), local therapy only	21	0.23 (0.06)*	0.11 to 0.34	3.93	51.14*	0.24 (0.10)*	0.03 to 0.44	2.26	16.62
Nguyen et al. (8), healthy comparison	21	−0.18 (0.06)*	−0.29 to −0.07	− 3.07	64.00*	−0.19 (0.10)	−0.39 to 0.02	−1.78	19.87
Schagen et al. (22), local therapy only	20	−0.28 (0.05)*	−0.38 to −0.18	− 5.41	17.48	−0.28 (0.10)*	−0.48 to −0.08	− 2.72	4.37
Scherwath et al. (26), high-dose chemotherapy vs. without chemotherapy	15	−0.03 (0.07)	−0.17 to 0.10	−0.47	12.37	−0.03 (0.12)	−0.28 to 0.21	−0.27	3.91
Scherwath et al. (26), standard-dose chemotherapy vs. without chemotherapy	15	−0.04 (0.07)	−0.18 to 0.09	−0.62	6.05	−0.04 (0.12)	−0.29 to 0.20	−0.35	1.93
Schilder et al. (23), healthy comparison	17	−0.27 (0.04)*	−0.36 to −019	− 6.25	24.20	−0.27 (0.11)*	−0.48 to −0.07	− 2.59	4.11
Shilling et al. (19), healthy comparison	8	−0.22 (0.07)*	−0.36 to −0.09	− 3.20	12.57	−0.22 (0.15)	−0.52 to 0.08	−1.46	2.61
Stewart et al. (18), hormonal therapy	22	0.04 (0.04)	−0.04 to 0.11	0.90	18.91	0.04 (0.09)	−0.15 to 0.22	0.39	3.41
van Dam et al. (5), high-dose chemotherapy vs. without chemotherapy	18	−0.27 (0.06)*	−0.38 to −0.16	− 4.80	47.20*	−0.27 (0.11)*	−0.49 to −0.06	− 2.48	12.75
van Dam et al. (5), standard-dose chemotherapy vs. without chemotherapy	18	−0.16 (0.06)*	−0.27 to −0.06	− 2.99	20.27	−0.17 (0.11)	−0.38 to 0.05	−1.52	5.24
Vearncombe et al. (17), without chemotherapy	13	0.13 (0.06)*	0.01 to 0.26	2.15	9.61	0.14 (0.13)	−0.11 to 0.38	1.07	2.38
Yamada et al. (9), healthy comparison	12	−0.39 (0.07)*	−0.54 to −0.25	− 5.40	26.54*	−0.40 (0.13)*	−0.67 to −0.14	− 2.98	7.85
				*Q* total	2519.48*			*Q* total	857.64*
				(df = 509)				(df = 509)	
				*Q* within	1943.48*			*Q* within	697.21*
				(df = 475)				(df = 475)	
				*Q* between	576.00*			*Q* between	160.43*
				(df = 33)				(df = 33)	

*p < 0.05.

Nevertheless, overall, in cross-sectional studies, patients treated with chemotherapy exhibited significantly worse cognitive functioning when contrasted with comparison groups, as shown in a small but significant grand weighted mean effect size of *d* = −0.12 (95% CIs from −0.14 to −0.11) using a fixed effects model, and *d* = −0.14 (95% CIs from −0.18 to −0.09) using a random effects model. However, tests of homogeneity were statistically significant for fixed effect (*Q*_Total_ = 2519.48, *p* < 0.05) and random effect (*Q*_Total_ = 857.64, *p* < 0.05) grand weighted mean effect size models, indicating that the sampled effect sizes were not derived from a single population.

### Prospective longitudinal studies

Weighted mean effect sizes for prospective longitudinal studies using fixed and random effect models are displayed in Table 4. Mean effect sizes ranged from −0.29 to 1.12 using the more conservative random effect model, with only two comparisons producing negative effect sizes representing worse cognitive functioning at follow-up compared to baseline assessments (34, 35). For prospective longitudinal studies, chemotherapy patients exhibited improved cognitive functioning from baseline (prior to chemotherapy) to follow-up (after chemotherapy) assessments, as shown in a small but significant grand weighted mean effect size of *d* = 0.11 (95% CIs from 0.09 to 0.14) using a fixed effects model, and *d* = 0.16 (95% CIs from 0.09 to 0.22) using a random effects model. However, tests of homogeneity were statistically significant for fixed effect (*Q*_Total_ = 1212.07, *p* < 0.05) and random effect (*Q*_Total_ = 615.63, *p* < 0.05) grand weighted mean effect size models, indicating that the sample of effect sizes were not derived from a single population. It is likely that the direction (i.e., better or worse cognitive functioning) and magnitude of effect sizes was partly dependent on the length of follow-up time (e.g., short vs. long follow-up), which is examined in subsequent moderator analyses.

**Table 4:** **Weighted mean effect sizes for prospective longitudinal studies**.

Study: Authors, reference, timing of follow-up	*k*	Fixed effect model				Random effect model			
		Effect size (SE)	95% CI	*z*	*Q*	Effect size (SE)	95% CI	*z*	*Q*
Grand mean weighted effect size	228	0.11 (0.01)*	0.09 to 0.14	8.78	1212.07*	0.16 (0.03)*	0.09 to 0.22	5.03	615.63*
Bender et al. (38), 1 week follow-up	18	1.02 (0.10)*	0.82 to 1.22	10.01	141.21*	1.12 (0.14)*	0.83 to 1.40	7.73	77.50*
Bender et al. (38),1 year follow-up	16	0.55 (0.13)*	0.30 to 0.79	4.36	266.88*	0.70 (0.16)*	0.38 to 1.03	4.30	181.48*
Collins et al. (33),1 year follow-up	23	0.21 (0.04)*	0.13 to 0.29	5.08	13.69*	0.21 (0.09)*	0.03 to 0.39	2.24	2.64
Collins et al. (33),1 month follow-up	23	0.10 (0.04)*	0.02 to 0.18	2.36	12.07	0.10 (0.09)	−0.09 to 0.28	1.03	2.31
Collins et al. (34), During chemotherapy	13	−0.22 (0.06)*	−0.34 to −0.10	− 3.58	191.60*	−0.26 (0.13)*	−0.51 to −0.00	− 1.98	182.66*
Debess et al. (37), 4 weeks follow-up	5	0.20 (0.07)*	0.06 to 0.34	2.75	2.94	0.20 (0.19)	−0.18 to 0.58	1.03	0.42
Hermelink et al. (25), between last second and last chemotherapy	12	0.20 (0.04)*	0.12 to 0.28	5.05	19.90	0.20 (0.12)	−0.04 to 0.45	1.67	2.15
Hurria et al. (16),6 months follow-up	13	0.05 (0.08)	−0.10 to.0.20	0.68	8.71	0.05 (0.14)	−0.21 to 0.32	0.40	2.79
Jansen et al. (35),6 months follow-up	7	−0.08 (0.07)	−0.22 to 0.05	−1.19	254.66*	−0.29 (0.17)	−0.62 to 0.04	−1.71	64.30*
Jenkins et al. (43),18 months follow-up	14	0.08 (0.04)	−0.00 to 0.16	1.95	19.79	0.08 (0.11)	−0.14 to 0.31	0.70	2.54
Jenkins et al. (43),4 weeks follow-up	14	0.03 (0.04)	−0.05 to 0.11	0.80	11.65	0.03 (0.11)	−0.19 to 0.26	0.29	1.49
Shilling et al. (19),6 months follow-up	9	0.05 (0.07)	−0.08 to 0.18	0.73	15.89	0.05 (0.15)	−0.24 to 0.34	0.33	3.19
Stewart et al. (18),2 months follow-up	23	0.12 (0.04)*	0.04 to 0.19	3.03	13.50	0.12 (0.09)	−0.06 to 0.30	1.28	2.37
Vearncombe et al. (17), 4 weeks follow-up	14	0.06 (0.03)*	0.00 to 0.13	1.98	66.04*	0.06 (0.11)	−0.16 to 0.28	0.57	5.67
Wefel et al. (27), 3 weeks follow-up	10	0.18 (0.11)	−0.03 to 0.38	1.67	2.47	0.18 (0.17)	−0.15 to 0.50	1.07	1.01
Wefel et al. (27), 1 year follow-up	10	0.26 (0.11)*	0.04 to 0.48	2.33	3.20	0.26 (0.17)	−0.07 to 0.59	1.55	1.41
Wefel et al. (27), 1 year follow-up	6	0.22 (0.10)*	0.02 to 0.42	2.16	15.66*	0.22 (0.19)	−0.16 to 0.60	1.14	4.33
				*Q* total (df = 228)	1212.07*			*Q* total (df = 228)	615.63*
				*Q* within (df = 211)	1059.84*			*Q* within (df = 211)	538.24*
				*Q* between (df = 16)	152.22*			*Q* between (df = 16)	77.39*

*p < 0.05.

For both cross-sectional and prospective longitudinal studies, there was significant variation within and across studies in the magnitude of effect sizes produced. This may suggest that other factors were impacting on the nature and magnitude of effect sizes within and across studies (e.g., type of neuropsychological measure, time since chemotherapy), with this being the focus of the remaining analyses.

### Cognitive domain

Effect sizes were grouped according to cognitive domain (i.e., attention, executive function, language, long-term memory, motor function, processing speed, short-term memory, and visuospatial function) for cross-sectional and prospective longitudinal studies using both fixed and random effect models (Table 5). There was variation in the magnitude of weighted mean effect sizes across cognitive domains, indicating that receiving chemotherapy was likely to be associated with specific rather than generalized cognitive effects.

**Table 5:** **Weighted mean effect sizes for cognitive domain**.

Cognitive domain	*k*	Fixed effect model				Random effect model			
		Effect size (SE)	95% CI	*z*	*Q*	Effect size (SE)	95% CI	*z*	*Q*
**Cross-sectional studies**
Attention	107	−0.13 (0.02)*	−0.18 to −0.09	−6.22	313.21*	−0.16 (0.05)*	−0.25 to −0.07	−3.49	74.93
Executive function	83	−0.16 (0.02)*	−0.21 to −0.12	−7.45	324.25*	−0.19 (0.05)*	−0.30 to −0.09	−3.72	62.57
Language	17	−0.04 (0.06)	−0.16 to 0.08	−0.60	42.25*	−0.08 (0.12)	−0.31 to 0.16	−0.64	14.68
Long-term memory	121	−0.08 (0.02)*	−0.12 to −0.04	−4.10	766.84*	−0.04 (0.04)	−0.13 to 0.05	−0.88	296.99*
Motor function	34	−0.11 (0.03)*	−0.17 to −0.05	−3.45	147.77*	−0.16 (0.08)*	−0.32 to −0.00	−1.98	55.54*
Processing speed	32	−0.23 (0.03)*	−0.29 to −0.16	−7.10	116.46*	−0.25 (0.08)*	−0.41 to −0.09	−3.04	19.41
Short-term memory	93	−0.11 (0.02)*	−0.15 to −0.07	−5.64	701.65*	−0.15 (0.05)*	−0.25 to −0.05	−3.04	296.20*
Visuospatial function	22	−0.02 (0.05)	−0.11 to 0.07	−0.48	80.98*	−0.06 (0.10)	−0.26 to 0.14	−0.55	28.02
				*Q* total (df = 509)	2519.48*			*Q* total (df = 509)	857.64*
				*Q* within (df = 501)	2493.41*			*Q* within (df = 501)	848.35*
				*Q* between (df = 7)	26.08*			*Q* between (df = 7)	9.29
**Prospective longitudinal studies**
Attention	52	0.12* (0.02)	0.07 to 0.17	4.88	60.53	0.12 (0.06)	−0.00 to 0.24	1.93	11.98
Executive function	37	0.08* (0.03)	0.02 to 0.13	2.56	58.83*	0.08 (0.07)	−0.06 to 0.28	1.11	10.17
Language	8	0.31* (0.08)	0.16 to 0.47	3.91	15.69*	0.26 (0.17)	−0.07 to 0.59	1.57	2.92
Long-term memory	55	0.22* (0.03)	0.17 to 0.28	8.29	333.63*	0.41* (0.06)	0.28 to 0.54	6.38	162.97*
Motor function	9	−0.10 (0.07)	−0.23 to 0.04	−1.44	33.68*	−0.00 (0.16)	−0.37 to 0.24	−0.41	6.38
Processing speed	7	0.14* (0.07)	0.01 to 0.28	2.12	7.19	0.12 (0.17)	−0.21 to 0.45	0.73	1.37
Short-term memory	51	0.06* (0.03)	0.01 to 0.12	2.22	482.73*	0.08 (0.07)	−0.05 to 0.22	1.24	340.84*
Visuospatial function	9	−0.18* (0.07)	−0.31 to −0.04	−2.53	164.60*	−0.29 (0.16)	−0.60 to 0.18	−1.85	50.41*
				*Q* total (df = 228)	1212.07*			*Q* total (df = 228)	615.63*
				*Q* within (df = 220)	1156.89*			*Q* within (df = 220)	587.04*
				Q Between (df = 7)	55.18*			Q between (df = 7)	28.59*

*p < 0.05.

For cross-sectional studies, weighted mean effect sizes ranged from −0.04 to −0.25 for cognitive domains using the more conservative random effects model. The largest effect sizes using the random effects model were found for the processing speed (*d* = −0.25) and executive function (*d* = −0.19) domains, indicating that when aggregating data across all studies, chemotherapy patients were more likely to experience impairments in these two domains relative to comparison groups. Weighted mean effect sizes for the cognitive domains of language, long-term memory and visuospatial function were not significantly different from zero using the random effects model, indicating that on average chemotherapy patients did not experience consistently marked impairments in these domains in contrast to comparison groups. Tests of homogeneity were statistically significant for fixed effect (*Q*_Total_ = 80.98, *p* < 0.05), but not for random effect (*Q*_Total_ = 28.02, *p* > 0.05) cognitive domain weighted mean effect size models. This provides some evidence that variation in effect sizes was reduced when taking cognitive domain into consideration for cross-sectional studies.

For prospective longitudinal studies, weighted mean effect sizes ranged from −0.29 to 0.41 for cognitive domains using the more conservative random effects model. Long-term memory was the only cognitive domain to produce a significant mean effect size (*d* = 0.41), indicating that chemotherapy patients typically exhibited improvements in long-term memory when re-assessed after baseline and after chemotherapy treatment had been completed. Tests of homogeneity were statistically significant for fixed effect (*Q*_Total_ = 1212.07, *p* < 0.05), and random effect (*Q*_Total_ = 615.63, *p* < 0.05) cognitive domain weighted mean effect size models. This indicated that variation in effect sizes remained even after taking cognitive domain into consideration for prospective longitudinal studies.

**Figure 2:**
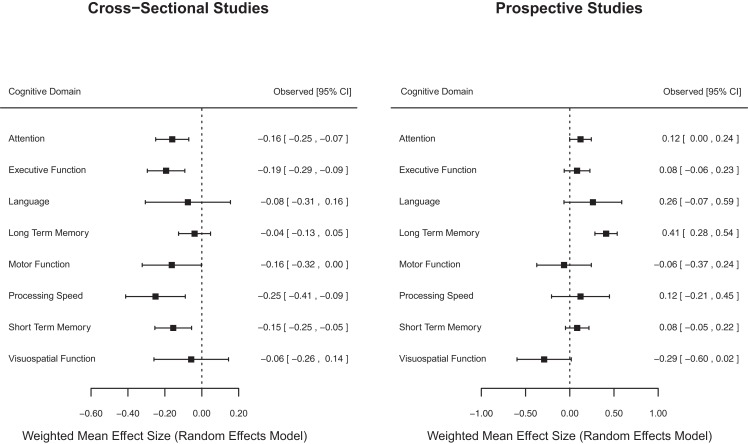
**Forest plot of cognitive domain weighted mean effect sizes for cross-sectional and prospective longitudinal studies**.

Figure 2 displays forest plots of weighted mean effect sizes for cognitive domains for cross-sectional and prospective longitudinal studies, where the mean effect size is represented by the marker, and the upper and lower 95% CIs for the estimate are represented by the horizontal lines connected to the marker. As shown in Figure 2, cross-sectional studies found a negative weighted mean effect size for all cognitive domains with a significant negative effect in five domains. On the other hand, prospective longitudinal studies found positive weighted mean effect sizes for most (6/8) cognitive domains with only one domain showing a significant positive mean effect size.

### Moderators

Meta-analytic regression was performed separately for cross-sectional and prospective longitudinal studies reporting data for the potential moderators of time since last chemotherapy treatment, average age when receiving chemotherapy, and comparison group type (healthy vs. patient controls) for cross-sectional studies only. For these analyses, mean study effect sizes were the dependent variable, and the inverse variance of mean effect sizes was used as the weighting variable. Displayed in Table 6 is a summary of the meta-analytic regression analyses for moderators using a random effects model.

**Table 6:** **Meta-analytic regression results for moderator variables**.

Variable	*B*	SE	*z*	95% CI
**Cross-sectional studies**
Intercept	0.75	0.90	0.83	−1.03 to 2.51
Comparison group	−0.52***	0.13	−4.02	−0.78 to −0.27
Age at treatment	0.02	0.01	1.82	−0.00 to 0.04
Time since final chemotherapy treatment	−0.00	0.00	−1.07	−0.00 to 0.00
Average years of education	−0.12*	0.05	−2.19	−0.22 to −0.01
*R*^2^ = 0.60				
*Q*_Model_ = 24.63*** (df = 4)				
*Q*_Residual_ = 89.49*** (df = 15)				
**Prospective longitudinal studies**
Intercept	1.25	1.05	1.19	−0.81 to 3.30
Age at treatment	−0.03**	0.01	−2.58	−0.06 to −0.01
Average years of education	0.04	0.07	0.59	−0.09 to 0.17
Time since final chemotherapy treatment	−0.00	0.00	−0.13	−0.00 to 0.00
*R*^2^ = 0.26				
*Q*_Model_ = 6.76 (df = 3)				
*Q*_Residual_ = 83.96*** (df = 8)				

*p < 0.05.

**p < 0.01.

***p < 0.001.

For cross-sectional studies, the *Q*_model_ was significant (*Q*_model_ = 24.63, df = 4, *p* < 0.001; *Q_Residual_* = 89.49, df = 15, *p* < 0.001), indicating that the moderator variables together accounted for a significant level of variability in effect sizes. The variables of comparison group and average years of education were significant moderators of mean study effect sizes. These results indicated that poorer performance on neuropsychological tests by chemotherapy patients (i.e., negative effect sizes) was associated with studies using healthy comparison groups (vs. patient comparisons), and chemotherapy patients with fewer years of education.

For prospective longitudinal studies, the *Q*_model_ was not significant (*Q*_model_ = 6.76, df = 3, *p* > 0.05; *Q_Residual_* = 83.96, df = 8, *p* < 0.001), indicating that the moderator variables together did not account for a significant level of variability in effect sizes. However, the variable of age at treatment emerged as a significant moderator, indicating that older age at chemotherapy treatment was associated with poorer performance on neuropsychological measures at follow-up.

For cross-sectional studies, effect sizes were calculated for IQ differences between chemotherapy and comparison groups for studies reporting such data, with negative effect sizes representing poorer intellectual functioning in chemotherapy groups. Study effect sizes (fixed effect model) ranged from -0.73 to 0.69, with the average weighted effect size for group differences in IQ being *d* = -0.02 (95% CIs from -0.10 to 0.07) across studies, indicating no significant difference between chemotherapy and comparison groups in IQ. Using meta-analytic regression (fixed effect model), mean IQ effect sizes were not significantly associated with mean study effect sizes for neuropsychological measures (*Q_Model_* = 1.47, df = 1, *p* > 0.05; *Q*_Residual_ = 491.16, df = 23, *p* < 0.001).

There were only six prospective longitudinal studies reporting data on IQ. Given this small sample size, no analyses were conducted to examine the association between IQ and effect size magnitude for prospective longitudinal studies.

### Publication bias

**Figure 3:**
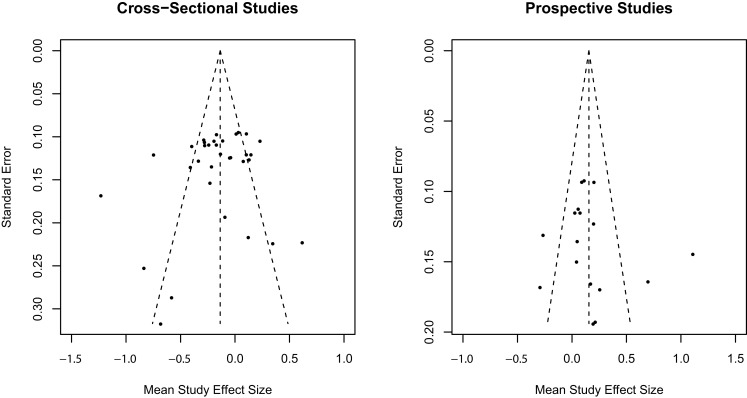
**Trim-and-fill analysis observed funnel plots for publication bias in mean study effect size**.

The trim-and-fill method (50) was used to assess publication bias separately for cross-sectional and prospective longitudinal studies using random effect model estimates. Inspection of the observed funnel plots of mean study effect sizes and the standard error of effect sizes in Figure 3 indicated symmetry around the overall weighted mean effect size suggestive of no significant publication bias for both cross-sectional and prospective longitudinal studies. Trim-and-fill analyses confirmed that no additional studies were required to adjust for an asymmetrical distribution of effect sizes for cross-sectional and prospective longitudinal studies.

## Discussion

In the current meta-analysis, 27 studies (*N* = 4361 participants) were reviewed and 737 effect sizes were generated to address two study aims: to examine the magnitude of cognitive impairment in eight cognitive domains and to identify factors moderating the magnitude of post-chemotherapy cognitive impairment among breast cancer patients. The findings generally indicated that the magnitude of cognitive impairment among chemotherapy groups varied within and across studies. Regardless, the grand mean weighted effect size suggests that subtle cognitive impairment was associated with adjuvant chemotherapy among breast cancer patients. This is consistent with previous meta-analyses (10, 11, 14, 15). The small but significant grand mean effect size may be due, partly, to varying levels of impairment across different cognitive domains and moderating factors not being taken into account. The mean effect sizes are discussed separately for each study design (cross-sectional vs. prospective).

### Study mean effect sizes by study design

#### Cross-sectional studies

Overall, breast cancer patients with a history of chemotherapy performed slightly, but significantly worse than their comparison groups. Nevertheless, 6 out of 34 comparisons from cross-sectional studies indicated that breast cancer patients previously treated with chemotherapy performed significantly better than individuals in the control group. However, these results were all based on comparisons of cognitive functioning between breast cancer patients with and without chemotherapy. Thus, these comparisons suggest that, generally, breast cancer patients previously treated with chemotherapy exhibit cognitive impairment as compared to their counterparts, but their impairment may not be worse than some breast cancer patients without chemotherapy.

#### Prospective longitudinal studies

In contrast, the grand mean effect size from the prospective longitudinal studies suggested that cognitive functioning among breast cancer patients treated with chemotherapy is slightly, but statistically significantly better after chemotherapy. However, this may not necessarily suggest that chemotherapy improves cognitive functioning or refutes the negative effects of chemotherapy on cognitive function. There are two other explanations, which are related to time since treatment and methodological limitations.

First, the results may be due to the timing of post-treatment assessment. It has been suggested that cognitive impairment associated with chemotherapy among breast cancer patients improves over time (10, 27, 43, 47). Follow-up assessment in these prospective longitudinal studies was conducted between 1–3 (33, 37, 38, 43) and/or 6–18 months (33, 35, 38, 43, 46) after last chemotherapy. Cognitive impairment at longer term follow-up may not be as marked as during or just after treatment. For example, breast cancer patients may have recovered from short-term cognitive impairment associated with chemotherapy and/or developed compensatory cognitive strategies after experiencing a series of chemotherapy doses. Given this possibility, time since treatment was examined as a moderating factor and is discussed later. However, some breast cancer patients treated with chemotherapy show long-term cognitive impairment (7), and a previous meta-analysis (11) did not find time since treatment to be a moderating factor. In addition, this does not explain why breast cancer patients’ cognitive functioning is better than (rather than equal to) pre-chemotherapy levels. Thus, other explanations need to be explored.

An alternative explanation relates to methodological issues inherent in prospective longitudinal studies. The first methodological issue that may have affected the results is potential practice effects on patients’ performance at follow-up. However, most prospective longitudinal studies included a method for managing practice effects. For example, a control group was employed to correct for practice effects (17, 25, 34) and/or alternative forms of tests were used at follow-up (27, 35, 38). Other studies employed a statistical correction for practice effects (18, 19, 27, 43). Regardless, practice effects were reported in studies that had employed alternative forms of tests at follow-up (35, 38). Indeed, only one study (34) reported significant post-chemotherapy cognitive decline among breast cancer patients. Furthermore, improved post-chemotherapy cognitive functioning was reported even when a control group was included (17, 25). These studies found improved post-chemotherapy cognitive functioning, even after quantifying and adjusting for practice effects based on improved performance in controls. Thus, practice effects may not fully explain improved post-chemotherapy cognitive function in patients.

The second methodological issue that may explain improved post-chemotherapy cognitive function relates to the timing of baseline assessment. More specifically, in all prospective longitudinal studies, patients’ baseline cognitive functioning was measured prior to chemotherapy, but either after diagnosis with and without some treatment (19, 25, 27, 35, 46, 47) or even after surgery (17, 18, 33, 34, 37, 38, 43). Consequently, the patients were aware of the presence of breast cancer, and some underwent a surgery or treatment, waiting for the commencement of chemotherapy. All but three studies in the current meta-analysis (19, 25, 47) either excluded breast cancer patients with psychiatric disorders, reported no significant group differences in psychological factors (fatigue, depression, and anxiety); or controlled for such factors. However, it is possible that emotional factors associated with a diagnosis of breast cancer (i.e., acute stress, depression) could negatively influence cognitive functioning for some individuals. Therefore, baseline data used in those studies may not be the same as patients’ pre-diagnosis baseline cognitive functioning. For instance, if chemotherapy patients’ post-diagnosis (i.e., pre-chemotherapy) performance was significantly worse than their pre-diagnosis baseline, their post-chemotherapy performance is likely to be better than their pre-chemotherapy performance. Then, even if their post-chemotherapy performance was much better than their post-diagnosis/pre-chemotherapy baseline, this may still be significantly worse than pre-diagnosis performance. Indeed, some studies have noted impaired performance in women with breast cancer prior to chemotherapy, in support of this explanation (25, 51). Therefore, the difference between pre- and post-chemotherapy cognitive performance in those studies may represent only a partial trajectory of post-chemotherapy cognitive functioning among breast cancer patients. This may in part explain improved post-chemotherapy cognitive function among breast cancer patients.

### Cognitive impairment by cognitive domains

#### Cross-sectional studies

It was found that breast cancer patients previously treated with chemotherapy performed significantly worse than (healthy or cancer) controls in the domains of attention, executive function, motor function, processing speed, and short-term memory. The level of cognitive function among chemotherapy patients in the domains of language, long-term memory, and visuospatial function was not significantly different from their counterparts. Of the previous meta-analyses, only Falleti et al. (10) analyzed effect sizes for cognitive domains by study design, and they found significant cognitive impairment in the domains of attention, executive function, motor function, verbal ability, visuospatial ability, and memory. Therefore, the current results are partially consistent with Falleti et al. (10). The inconsistency may be partly due to an increased number of comparisons included in the current meta-analysis. It should be noted that the level of heterogeneity of cross-sectional studies was non-significant using a random effect model when studies were analyzed by cognitive domains. This supports the validity of the current results and suggests that the magnitude of post-chemotherapy cognitive impairment among breast cancer patients varies, depending on the cognitive domain.

#### Prospective longitudinal studies

In contrast, no post-chemotherapy cognitive decline was found among breast cancer patients in prospective longitudinal studies. Instead, breast cancer patients showed significantly improved long-term memory after chemotherapy. Although Falleti et al. (10) found post-chemotherapy cognitive improvement among breast cancer patients, they included only one study. Thus, there is no previous review to allow comparison with the current results. In addition, as discussed previously, issues regarding the varied timing of post-chemotherapy assessment, practice effects, and post-diagnosis baseline need to be considered in the interpretation of results from prospective longitudinal studies.

The cognitive domains (except visuospatial function) that showed less impairment in cross-sectional studies were also those more likely to show greater improvement in prospective longitudinal studies. For example, long-term memory was found to be least impaired in cross-sectional studies and was found to be the domain most likely to improve in prospective longitudinal studies. Although hypothetical, long-term memory may be the cognitive domain that is less likely to be affected by chemotherapy and/or is more likely to improve faster than other domains. Alternatively, it is possible that measures of long-term memory may be more susceptible to practice effects.

Language and visuospatial function have previously been reported as the most impaired cognitive domains among breast cancer patients treated with chemotherapy (10, 11, 14, 15). However, the magnitude of post-chemotherapy cognitive impairment and cognitive decline in language and visuospatial function among breast cancer patients was non-significant in this review. The discrepancy in findings between previous meta-analyses and this analysis may be due to an increased number of comparisons included in this study. More specifically, the results in previous meta-analyses were derived from a small number of comparisons (*k* = 3–15 for language and *k* = 5-10 for visuospatial function). Whereas in the present meta-analysis a larger number of comparisons was included, the domains were examined separately for cross-sectional studies (*k* = 17 for language and *k* = 22 for visuospatial function) and for prospective longitudinal studies (*k* = 8 for language and *k* = 9 for visuospatial function). Based on the large number of comparisons and separate analyses by study design, these domains were found to be non-significant. It should be noted that the CIs of the grand mean effect sizes for language and visuospatial function varied widely, and the non-significant results may be due to variability across studies in the results on these domains.

### Effects of moderators

#### Cross-sectional studies

Among cross-sectional studies, type of control group was found to significantly moderate the magnitude of cognitive impairment. More specifically, level of cognitive functioning among breast cancer patients with a history of chemotherapy was significantly worse than healthy controls, but not significantly worse than breast cancer patients without chemotherapy. In addition, level of education was found to significantly moderate the magnitude of post-chemotherapy cognitive impairment. That is, chemotherapy patients with lower levels of education tend to show greater cognitive impairment than those with higher levels of education. However, time since treatment and age at treatment were not significant moderators. These results contrast with those of Falleti et al. (10) but are partially consistent with Jim et al. (11) who found non-significant moderating effects of time since treatment and age. The meta-analysis by Falleti et al. (10) was based on only six cross-sectional studies and did not include type of control group as a moderator, which was found to be the most significant factor in the current review. These differences may explain the inconsistent findings. The main findings arising from the cross-sectional studies are that significantly greater cognitive impairment is observed among breast cancer patients previously treated with chemotherapy when compared to healthy controls, and that lower education level may be a risk factor for cognitive impairment. However, it is further noteworthy that levels of cognitive impairment are similar among breast cancer patients, irrespective of a history of chemotherapy.

#### Prospective longitudinal studies

Conversely, in prospective longitudinal studies older age was associated with increased levels of cognitive decline among breast cancer patients previously treated with chemotherapy. Falleti et al. (10) found the opposite results, with younger breast cancer patients exhibiting greater cognitive impairment after chemotherapy. However, their results were based on cross-sectional studies only and thus cannot be directly compared to the present findings. The current review suggests that cognitive decline associated with chemotherapy for breast cancer may interact with age, whereby older patients may have a higher risk of developing and/or experiencing persisting cognitive decline after chemotherapy. The negative effects of older age on cognitive function are well documented (52), including cognitive decline in the domains of processing speed, attention and executive function. Thus, it is possible that chemotherapy exacerbates the effects of old age on cognitive function for breast cancer patients.

### Strengths and limitations of current review

The current meta-analysis extended upon previous reviews to improve understanding of the effects of chemotherapy on cognitive functioning among breast cancer patients. The results were based on good search strategy and a larger number of studies that employed validated neuropsychological measures. Indeed, the results of the publication bias analysis supported the validity of the findings. In addition, study design (e.g., cross-sectional vs. prospective) has been suggested to moderate the results (10, 11). To address this issue, the grand mean effect sizes and meta-regression analyses of moderators were conducted for cross-sectional studies and prospective longitudinal studies.

Regardless of these strengths, some limitations need to be acknowledged, many of which are inherent in meta-analyses. First, as suggested in *Q* statistics, the effect sizes varied significantly across studies, and studies were heterogeneous with respect to many factors, such as the measures used, participants’ characteristics, cancer stages, type and dosage of chemotherapy and hormone therapy, time since therapy, and control type. Therefore, whether these factors moderate the results is yet to be examined. In addition, although the type of control group was found to significantly moderate the magnitude of post-chemotherapy cognitive impairment, other potential moderators, such as type and dosage of chemotherapy and the current use of tamoxifen, were not included in this review.

### Future directions

As discussed above, it is important to examine other factors that potentially moderate the magnitude of post-chemotherapy cognitive impairment/declining, especially over the long-term. First, it is still uncertain whether the use of tamoxifen itself, or the interaction between tamoxifen and chemotherapy, leads to the development of and/or persistence of cognitive impairment among breast cancer patients. This question is not new, yet the findings of previous meta-analyses have been mixed (10, 11). Second, it also remains unclear whether or not level of cognitive performance at pre-chemotherapy (but post-diagnosis) is the same as that at pre-diagnosis. To answer these research questions, a prospective longitudinal study needs to be conducted, in which cognitive functioning is compared between four groups: healthy controls; breast cancer patients with chemotherapy only (and no hormone therapy); patients with chemotherapy and hormone therapy; and patients with hormone therapy only. Cognitive functioning should be measured prior to diagnosis (e.g., at regular screening examinations), as well as just before, during, and after chemotherapy. This type of study would also answer another research question that emerged from this review – whether or not the effects of chemotherapy on cognitive functioning are worse than those of other treatments. However, this type of study may not be easily conducted, and conducting a cross-sectional study with an improved study design would still be helpful. For example, comparing cognitive functioning between the following groups may identify the moderating factors: a matched healthy control group, a matched cancer control group (diagnosed, but not treated), and treated breast cancer groups (surgery only, chemotherapy only, chemotherapy and hormonal therapy, and hormonal therapy only).

No consistent association between psychological factors (i.e., depression, anxiety, or fatigue) and performance on objective measures of cognitive functioning has been found (7, 8). Some studies have even reported that depression and fatigue were significantly related to subjective, but not objective cognitive complaints (5, 36). However, the lack of association between psychological factors and post-chemotherapy cognitive impairment among breast cancer patients may be due partly to the issue of ecological validity of the objective measures (42). In addition, breast cancer patients with depression have typically been excluded from studies (5, 8, 17, 18, 20–24, 27, 31–36), or these factors were statistically controlled for (7, 26, 38). It is also possible that depressed breast cancer patients are less likely to participate in research. Hence, the relationship between mental health issues and post-chemotherapy cognitive impairment remains unclear, and this needs to be examined in future research.

Finally, it may be important to measure additional cognitive domains. For example, further subdivision of some cognitive domains may help identifying specific cognitive functions that are vulnerable to the process (diagnosis, treatment, and recovery) of breast cancer and, this would consequently help clinicians providing patients with focused intervention. More specifically, executive function may be subdivided into working memory, inhibition, and shifting, while attention may be subdivided into attention span, selective attention, and focused attention. Furthermore, an investigation of cognitive domains that have not included in previous studies would also be beneficial. For instance, impairments in prospective memory, or the ability to remember what to do in future, would have significant clinical implications.

## Conclusion

The effects of chemotherapy on cognitive functioning among breast cancer patients were found to be subtle, but relatively global with five of eight domains being impaired. These findings indicate that some cognitive domains are more (e.g., processing speed) or less (e.g., long-term memory) susceptible to chemotherapy than others. Further, particular cognitive domains (e.g., long-term memory) may show greater improvement over time than others albeit these domains may be susceptible to practice effects. Because individuals’ levels of cognitive performance at pre-chemotherapy assessment may not be the same as their pre-diagnosis performance, it remains unclear whether, and to what degree post-chemotherapy cognitive decline in breast cancer patients improves or persists. A significant level of cognitive impairment was observed in breast cancer patients previously treated with chemotherapy, as compared to healthy controls. However, level of cognitive impairment in chemotherapy patients did not significantly differ from breast cancer patients without chemotherapy. Hence, cognitive impairment may be common among breast cancer patients irrespective of their treatment regimens. Furthermore, patient characteristics (age and educational level) and the processes of cancer diagnosis and treatment may moderate the magnitude of cognitive impairment. This is the first review that examined and found the moderating effect of the type of control groups in cross-sectional studies. Future prospective longitudinal research is warranted to examine the degree and persisting nature of cognitive impairment present after chemotherapy, with comparisons made to participants’ cognitive function *prior to diagnosis*. Accurate understanding of the effects of chemotherapy is essential to enable informed decisions regarding treatment and to improve quality of life among breast cancer patients.

## Conflict of Interest Statement

The authors declare that the research was conducted in the absence of any commercial or financial relationships that could be construed as a potential conflict of interest.
